# Novel Pyrimidine Derivatives Bearing a 1,3,4-Thiadiazole Skeleton: Design, Synthesis, and Antifungal Activity

**DOI:** 10.3389/fchem.2022.922813

**Published:** 2022-06-08

**Authors:** Nianjuan Pan, Chunyi Liu, Ruirui Wu, Qiang Fei, Wenneng Wu

**Affiliations:** Food and Pharmaceutical Engineering Institute, Guiyang University, Guiyang, China

**Keywords:** 4-thiadiazole, pyrimidine, design, synthesis, antifungal activity

## Abstract

In this study, twenty novel pyrimidine derivatives bearing a 1,3,4-thiadiazole skeleton were designed and synthesized. Then their antifungal activity against *Botrytis cinereal* (*B. cinereal*), *Botryosphaeria dothidea* (*B. dothidea*), and *Phomopsis* sp. were determined using the poison plate technique. Biological test results showed that compound **6h** revealed lower EC_50_ values (25.9 and 50.8 μg/ml) on *Phompsis* sp. than those of pyrimethanil (32.1 and 62.8 μg/ml).

## 1 Introduction

Due to their structure, which is similar to their alkaloid-like structure in living organisms, nitrogen-containing heterocyclic compounds have the characteristics of high target specificity and good environmental compatibility and have become the mainstream research field for the creation of new pesticides (Li et al., 2017; He et al., 2019). Among them, 1,3,4-thiadiazoles containing both N and S elements in the heterocyclic structure are important and lead molecules for designing biologically active compounds with various biological activities (Hu et al., 2014). For the past years, a large number of studies have shown that 1,3,4-thiadiazole and their derivatives had various biological activities including herbicidal (Sun et al., 2013), bactericidal (Li et al., 2015; Zhang et al., 2019; Wu Q. et al., 2020; Wu et al., 2021), fungicidal (Zou et al., 2002; Zine et al., 2016; Wu W. et al., 2020), antiviral (Wu et al., 2016a; Gan et al., 2017), insecticidal (Dai et al., 2016; Lv et al., 2018), anticancer (Chen et al., 2019), and so on. In the field of medicine and pesticides, especially in the field of fungicides, the products that have been successfully developed at present are thiabendazole, thiabendron copper, thiazole zinc, and thiazole.

Meanwhile, in the agricultural field, pyrimidine derivatives also have good biological activities such as antiviral (Wu, et al., 2015; Zan et al., 2020), insecticidal (Liu, et al., 2017; Wu, et al., 2019; Chen, et al., 2021; Liu, et al., 2021; Sun, et al., 2021), fungicidal (Guan et al., 2017; Yan et al., 2020; Yang, et al., 2020), bactericidal (Li et al., 2020), herbicidal (Chen et al., 2019; Li et al., 2020), and anticancer (Guo et al., 2020) properties. In the last few decades, some pyrimidine derivatives have been commercialized as pesticides for controlling plant diseases and insect pests. Therefore, pyrimidine was considered an active substructure to develop promising pesticides in recent years.

**FIGURE 1:**
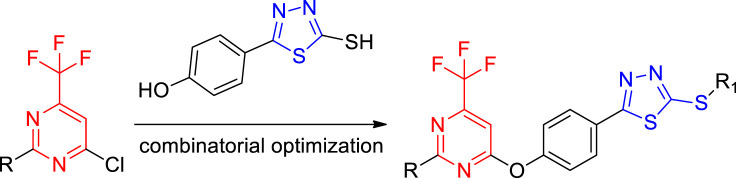
Design of the target compounds.

Based on the biological activity of 1,3,4-thiadiazole and the pyrimidine ring, in order to find new pyrimidine lead compounds with good biological activity, this work adopts the active substructure splicing method to design and synthesize a series of novel pyrimidine derivatives containing a 1,3,4-thiadiazole moiety (Figure 1), which were evaluated *in vitro* with regard to their antifungal activity against *Botrytis cinereal* (*B. cinereal*), *Botryosphaeria dothidea* (*B. dothidea*), and *Phomopsis* sp.

## 2 Materials and Methods

### 2.1 Chemistry

Melting points (m.p.) were obtained using a microscope apparatus (XT-4, Beijing Tech Instrument Co., China). Nuclear magnetic resonance (^1^H NMR and ^13^C NMR) was determined on a Bruker NMR spectrometer (Bruker, Germany). High-resolution mass spectrometry (HRMS) was performed on a Thermo Scientific Q Exactive Plus instrument (Thermo Fisher Scientific, United States).

### 2.2 The Preparation Procedure of Intermediates 1–5

Intermediates **1** and **2** were obtained by referring to the previously reported methods (Wu W. et al., 2020).

**SCHEME 1:**
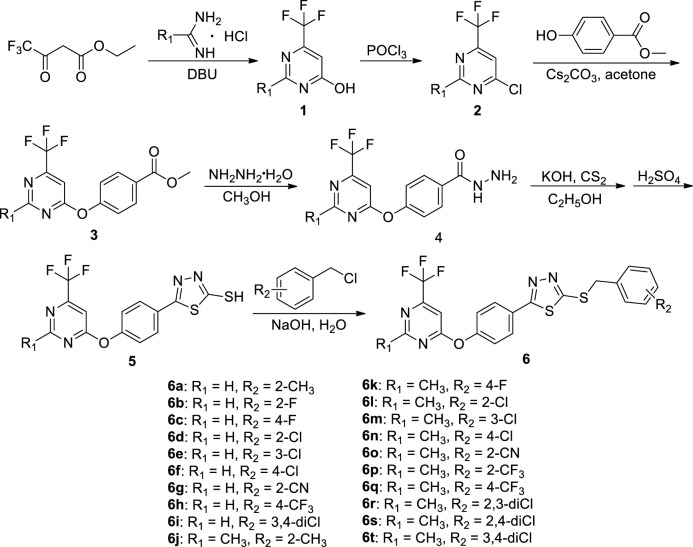
Synthetic process and experimental method of the target compounds **6a**−**6t**.

To a 100-ml three round-bottom flask, intermediate **2** (0.01 mol), ethyl 4-hydroxybenzoate (0.012 mol), Cs_2_CO_3_ (0.02 mol), and acetone (50 ml) were added. After reacting for 2–4 h at room temperature, the solvent was vacuum evaporated. The residues were recrystallized from ethanol to give pure intermediate **3**.

To a solution of intermediate **3** (20 mmol) in 40 ml absolute methanol, 80% hydrazine hydrate (60 mmol) was added dropwise. After reacting for 5–7 h under reflux conditions, the reaction was quenched to room temperature. The white solids precipitated from the reaction solution were filtrated and recrystallized from ethanol to give pure intermediate **4**.

To a mixture of intermediate **4** (30 mmol), KOH (45 mmol), and ethanol (500 ml), carbon disulfide (36 mmol) was added dropwise. The white precipitates were filtered, dried under vacuum, and then added to 30 ml precooled concentrated H_2_SO_4_. After stirring for 2 h at 0°C, the mixture was poured into 1,000 ml ice water and neutralized with sodium bicarbonate saturated solution (Wu et al., 2016a; Wu et al., 2016b). The filtrate was acidified with 5% hydrochloric acid, and the produced solid was filtered and recrystallized from ethanol to give the key intermediate **5**.

### 2.3 Preparation Procedure of the Target Compounds 6a−6t

Intermediate **5** (2 mmol), NaOH (2.2 mmol) dissolved in 15 ml water, and substituted benzyl chloride (2.1 mmol) were added in a 100-ml three round-bottom flask and stirred at room temperature for 2–4 h (Scheme 1). Upon completion of reaction, the residues were filtered and recrystallized from ethanol to produce the pure target compounds **6a**–**6t**. The physical properties, NMR, and HRMS for title compounds are reported in Supplementary Data S1, and the spectral data of **6a** are shown below. 2-((2-methylbenzyl)thio)-5-(4-((6-(trifluoromethyl)pyrimidin-4-yl)oxy)phenyl)-1,3,4-thiadiazole (**6a**). White solid; yield 65.24%; m. p. 104–107°C; ^1^H NMR (600 MHz, DMSO-*d*_*6*_, ppm) *δ*: 8.99 (s, 1H, pyrimidine-H), 8.04–8.02 (m, 2H, phenyl-H), 7.86 (s, 1H, pyrimidine-H), 7.50–7.48 (m, 4H, phenyl-H), 7.42 (d, 1H, *J* = 5.4 Hz, phenyl-H), 7.23–7.17 (m, 3H, phenyl-H), 4.65 (s, 2H, -SCH_2_-), 2.41 (s, 3H, pyrimidine-CH_3_); ^13^C NMR (150 MHz, DMSO-*d*_*6*_, ppm) *δ*: 170.32, 167.66, 165.34, 159.73, 156.22 (q, *J* = 35.1 Hz), 154.29, 137.37, 134.12, 130.98, 130.59, 129.74, 128.65, 127.66, 126.62, 123.27, 121.80 (q, *J* = 272.7 Hz), 116.13, 107.07, 36.66, 19.26; HRMS (ESI) calcd for C_21_H_15_ON_4_S_2_F_3_ [M+Na]^+^: 483.05249, found: 483.05316.

### 2.4 *In vitro* Antifungal Activity Test

The *in vitro* antifungal activity was determined according to the mycelial growth rate method (Zhang et al., 2018; Wang et al., 2019; Wu Q. et al., 2020). Each target compound (5 mg) was dissolved in DMSO (1 ml) and added to 9 ml H_2_O and 90 ml potato dextrose agar (PDA) medium to prepare 9 dishes of mixed PDA plates with a concentration of 50 μg/ml. After that, a 0.4-cm diameter of each test fungus was put onto the middle of mixed PDA plates and fostered in an incubator at 28°C for 3–4 days. After the mycelia diameter of the untreated PDA plate reached 5–6 cm, the inhibition rates *I* (%) are calculated using the following formula, where C (cm) and T (cm) represent the fungi diameters of the untreated and treated PDA plates, respectively.

Inhibition rate I (%)=(C−T)/(C−0.4)×100

## 3 Results and Discussion

### 3.1 Chemistry

In the ^1^H NMR data of compound **6a**, a singlet appears at 4.65 ppm and indicates the presence of the -SCH_2_- group. The CH proton of the 6-trifluoromethylpyrimidine ring appeared as two singlets at 8.99 and 7.86 ppm. Meanwhile, in the ^13^C NMR data of compound **6a**, two signals at 170.32 and 167.66 ppm indicated the presence of C proton in the 1,3,4-thiadiazole group. One quartet at 156.22 ppm indicated the presence of -CF_3_ in the pyrimidine fragment. In addition, compound **6a** was confirmed correctly by combining HRMS data with the [M + Na]^+^ peaks.

### 3.2 *In vitro* Antifungal Activity

As shown in Table 1, compounds **6c**, **6g**, and **6h** exhibited higher *in vitro* antifungal activity against *Phomopsis* sp., and the inhibition rates were 89.6%, 88.7%, and 89.2%, respectively, compared to that of pyrimethanil (85.1%). Meanwhile, Table 1 shows that the inhibitory activity values of compounds **6g**, **6h**, and **6q** against *B. cinerea* were 86.1%, 90.7%, and 88.3%, respectively, which were superior to that of pyrimethanil (82.8%). In addition, compound **6h** possessed similar bioactivity against *B. dothidea* (82.6%) to that of pyrimethanil (84.4%).

**TABLE 1:** Inhibition rates of compounds **6a−6t** against *B. cinereal*, *B. dothidea*, and *Phomopsis* sp. at 50 µg/ml.

Compounds	Inhibition rate (%)		
	*B. dothidea*	*Phomopsis* sp.	*B. cinerea*
**6a**	41.8 ± 2.1	50.6 ± 2.2	73.2 ± 1.8
**6b**	63.0 ± 1.3	83.2 ± 1.3	78.7 ± 1.3
**6c**	75.6 ± 1.1	89.6 ± 1.8	85.1 ± 2.5
**6d**	57.4 ± 1.5	74.6 ± 1.4	71.1 ± 1.9
**6e**	65.9 ± 1.3	79.4 ± 2.1	79.2 ± 2.3
**6f**	72.4 ± 2.6	84.5 ± 1.2	84.9 ± 2.4
**6g**	80.0 ± 1.9	88.7 ± 2.2	86.1 ± 3.2
**6h**	82.6 ± 2.6	89.2 ± 1.9	90.7 ± 2.6
**6i**	70.8 ± 1.1	84.6 ± 1.2	85.4 ± 1.1
**6j**	36.2 ± 3.0	42.9 ± 2.1	65.3 ± 1.4
**6k**	59.0 ± 1.0	71.6 ± 1.8	74.0 ± 1.8
**6l**	51.5 ± 1.2	64.5 ± 1.7	65.7 ± 1.2
**6m**	57.4 ± 1.7	71.9 ± 1.3	73.3 ± 1.2
**6n**	65.4 ± 2.3	78.4 ± 1.4	80.4 ± 2.4
**6o**	73.7 ± 3.3	76.7 ± 1.0	78.8 ± 2.6
**6p**	68.4 ± 1.8	80.3 ± 1.5	81.8 ± 1.2
**6q**	75.7 ± 1.9	86.8 ± 1.9	88.3 ± 0.9
**6r**	58.2 ± 1.5	69.0 ± 1.7	66.5 ± 1.3
**6s**	75.6 ± 1.6	82.4 ± 1.4	83.9 ± 2.2
**6t**	65.7 ± 1.7	78.0 ± 1.3	80.8 ± 1.5
Pyrimethanil	84.4 ± 2.1	85.1 ± 1.4	82.8 ± 1.4

Table 2 shows that compounds **6c**, **6g**, and **6h** had the EC_50_ values of 25.4, 28.8, and 25.9 μg/ml, respectively, which were better than that of pyrimethanil (32.1 μg/ml). Meanwhile, compounds **6g** (EC_50_ = 57.5 μg/ml) and **6h** (EC_50_ = 50.8 μg/ml) exhibited better *in vitro* bioactivity on *B. cinerea* than pyrimethanil (62.8 μg/ml). Meanwhile, compounds **6g** (EC_50_ = 67.8 μg/ml) and **6h** (EC_50_ = 63.6 μg/ml) exhibited lower *in vitro* bioactivity against *B. dothidea* than pyrimethanil (57.6 μg/ml).

**TABLE 2:** EC_50_ values of the title compounds against *B. dothidea*, *Phomopsis* sp., and *B. cinereal*.

Compounds	EC_50_ (μg/ml)		
	*B. dothidea*	*Phomopsis* sp.	*B. cinerea*
**6c**	—	25.4 ± 2.3	63.2 ± 1.2
**6f**	—	37.5 ± 1.7	67.6 ± 1.5
**6g**	67.8 ± 1.3	28.8 ± 2.6	57.5 ± 1.3
**6h**	63.6 ± 1.8	25.9 ± 1.4	50.8 ± 2.7
**6i**	—	34.8 ± 1.9	64.1 ± 2.9
**6q**	—	32.6 ± 1.5	59.9 ± 1.1
**6s**	—	—	68.8 ± 2.4
Pyrimethanil	57.6 ± 1.8	32.1 ± 2.0	62.8 ± 1.7

Further structure–activity relationship analysis indicated that more than 80% of the title compounds showed excellent antifungal activity against *Phomopsis* sp. and *B. cinerea*. Meanwhile, changing R_1_ (H or CH_3_) did not significantly improve the antifungal activity of the compound. Only against *Phomopsis* sp., the number of compounds (R_1_ = H) with activity higher than 80% is twice that of compounds (R_1_ = CH_3_). In addition, the introduction of strong electron withdraw groups (CN and CF_3_) into R_2_ was able to enhance the activity of the compounds, while the introduction of an alkyl group (CH_3_) cannot obviously improve the antifungal activity of the compounds.

## 4 Conclusion

In conclusion, 20 novel 1,3,4-thiadiazole derivatives bearing a pyrimidine skeleton were synthesized and assessed for all compounds with regard to *in vitro* antifungal activities. Results of bioassays of the synthesized compounds showed excellent antifungal activity compared to that of pyrimethanil. Therefore, 1,3,4-thiadiazole derivatives bearing a pyrimidine skeleton can be used as candidate leading structures for discovering new fungicidal agents.

## Data Availability

The original contributions presented in the study are included in the article/Supplementary Material; further inquiries can be directed to the corresponding author.
