# Supplementary material for: Novel Pyrimidine Derivatives Bearing a 1,3,4-Thiadiazole Skeleton: Design, Synthesis, and Antifungal Activity
Source: Front Chem. 2022 Jun 8;10:922813. doi: 10.3389/fchem.2022.922813 (PMC9213879; doi:10.3389/fchem.2022.922813)
Supplement: Supplementary file 1 [file DataSheet1.DOCX]

Novel Pyrimidine Derivatives Bearing an 1,3,4-Thiadiazole Skeleton: Design, Synthesis, and Antifungal Activity

Nianjuan Pan^#^, Chunyi Liu^#^, Ruirui Wu, Qiang Fei, Wenneng Wu*

Food and Pharmaceutical Engineering Institute, Guiyang University, Guiyang 550025, China

**Supplementary data**

**^1^H NMR spectral data for synthesized compounds 1-5**

Data for methyl 4-((6-(trifluoromethyl)pyrimidin-4-yl)oxy)benzoate (**3a**). White soild; yield 70.42%; m.p. 125–126 °C; ^1^H NMR (600 MHz, DMSO-*d_6_*, ppm) *δ*: 9.03 (s, 1H, pyrimidine-H), 8.42 (s, 1H, pyrimidine-H), 8.23 (d, 2H, *J* = 8.40 Hz, phenyl-H), 7.85 (d, 2H, *J* = 8.40 Hz, phenyl-H), 3.87 (s, 3H, -COOCH_3_).

yield 70.05%; m.p. 108–111 °C; ^1^H NMR (600 MHz, DMSO-*d_6_*, ppm) δ: 8.06 (d, 2H, *J* = 9.00 Hz, phenyl-H), 7.58 (s, 1H, pyrimidine-H), 7.42 (d, 2H, *J* = 9.00 Hz, phenyl-H), 3.87 (s, 3H, -COOCH_3_), 2.51 (s, 3H, pyrimidine-CH_3_).

Data for 4-((6-(trifluoromethyl)pyrimidin-4-yl)oxy)benzohydrazide (**4a**). White soild; yield 82.50%; m.p. 165–166 °C; ^1^H NMR (600 MHz, DMSO-*d_6_*, ppm) *δ*: 9.55 (s, 1H, -CONH-), 9.03 (s, 1H, pyrimidine-H), 8.42 (s, 1H, pyrimidine-H), 8.23 (d, 2H, *J* = 8.40 Hz, phenyl-H), 7.85 (d, 2H, *J* = 8.40 Hz, phenyl-H), 5.24 (s, 2H, -CONH-NH_2_).

Data for 4-((2-methyl-6-(trifluoromethyl)pyrimidin-4-yl)oxy)benzohydrazide (**4b**). White soild; yield 78.5%; m.p. 152–154 ^o^C; ^1^H NMR (600 MHz, DMSO-*d_6_*, ppm) *δ*: 9.52 (s, 1H, -CONH-),8.06 (d, 2H, *J* = 9.00 Hz, Ph-H), 7.58 (s, 1H, pyrimidine-H), 7.42 (d, 2H, *J* = 9.00 Hz, phenyl-H), 5.22 (s, 2H, -CONH-NH_2_), 2.51 (s, 3H, pyrimidine-CH_3_).

Data for 5-(4-((6-(trifluoromethyl)pyrimidin-4-yl)oxy)phenyl)-1,3,4-thiadiazole-2-thiol (**5a**). White soild; yield 65.24%; m.p. 198–199 °C; ^1^H NMR (600 MHz, DMSO-*d_6_*, ppm) δ: 9.04(s, 1H, pyrimidine-H), 8.44(s, 1H, pyrimidine-H), 8.20 (d, 2H, *J* = 8.40 Hz, phenyl-H), 7.83(d, 2H, *J* = 8.40 Hz, phenyl-H).

Data for 5-(4-((2-methyl-6-(trifluoromethyl)pyrimidin-4-yl)oxy)phenyl)-1,3,4-thiadiazole-2-thiol (**5b**). White soild; yield 70.8%; m.p. 184–185 °C; ^1^H NMR (600 MHz, DMSO-*d_6_*, ppm) δ: 8.08 (d, 2H, *J* = 9.0 Hz, phenyl-H), 7.59 (s, 1H, pyrimidine-H), 7.45 (d, 2H, *J* = 9.0 Hz, phenyl-H), 2.52 (s, 3H, pyrimidine-CH_3_).

**^1^H NMR, ^13^C NMR and HRMS spectral data for synthesized compounds**

Data for 2-((2-methylbenzyl)thio)-5-(4-((6-(trifluoromethyl)pyrimidin-4-yl)oxy)

phenyl)-1,3,4-thiadiazole (**6a**). White solid; yield 65.24%; m. p. 104−107 °C; ^1^H NMR (600 MHz, DMSO-*d_6_*, ppm) δ: 8.99 (s, 1H, pyrimidine-H), 8.04−8.02 (m, 2H, phenyl-H), 7.86 (s, 1H, pyrimidine-H), 7.50−7.48 (m, 4H, phenyl-H), 7.42 (d, 1H, *J* = 5.4 Hz, phenyl-H), 7.23−7.17 (m, 3H, phenyl-H), 4.65 (s, 2H, -SCH_2_-), 2.41 (s, 3H, pyrimidine-CH_3_); ^13^C NMR (150 MHz, DMSO-*d_6_*, ppm) δ: 170.32, 167.66, 165.34, 159.73, 156.22 (q, *J* = 35.1 Hz), 154.29, 137.37, 134.12, 130.98, 130.59, 129.74, 128.65, 127.66, 126.62, 123.27, 121.80 (q, *J* = 272.7 Hz), 116.13, 107.07, 36.66, 19.26; HRMS (ESI) calcd for C_21_H_15_ON_4_S_2_F_3_ [M+Na]^+^: 483.05249, found: 483.05316.

Data for 2-((2-fluorobenzyl)thio)-5-(4-((6-(trifluoromethyl)pyrimidin-4-yl)oxy)

phenyl)-1,3,4-thiadiazole (**6b**). White solid; yield 56.85%; m.p. 142−145 °C; ^1^H NMR (600 MHz, DMSO-*d_6_*, ppm) δ: 8.99 (s, 1H, pyrimidine-H), 8.04 (d, 2H, *J* = 8.40 Hz, phenyl-H), 7.86 (s, 1H, pyrimidine-H), 7.59−7.56 (m, 1H, phenyl-H), 7.50 (d, 2H, *J* = 8.40 Hz, phenyl-H), 7.40−7.36 (m, 1H, Ph-H), 7.26 (t, 1H, *J* = 9.6 Hz, phenyl-H), 7.21−7.18 (m, 1H, phenyl-H), 4.66 (s, 2H, -SCH_2_-); ^13^C NMR (150 MHz, DMSO-*d_6_*, ppm) δ: 170.32, 167.96, 164.81, 161.77, 160.13, 159.73, 156.22 (q, *J* = 34.8 Hz), 154.32, 132.01, 130.68, 129.78, 127.60, 125.09, 123.89, 123.37, 121.78 (q, *J* = 273.45 Hz), 116.09, 107.07, 31.83; HRMS (ESI) calcd for C_20_H_12_ON_4_S_2_F_4_ [M+Na]^+^: 487.02725, found: 487.02809.

Data for 2-((4-fluorobenzyl)thio)-5-(4-((6-(trifluoromethyl)pyrimidin-4-yl)oxy)

phenyl)-1,3,4-thiadiazole (**6c**). White solid; yield 72.43%; m.p. 133−135 °C; ^1^H NMR (600 MHz, DMSO-*d*_6_, ppm) δ: 8.99 (s, 1H, pyrimidine-H), 8.03 (d, 2H, *J* = 8.40 Hz, phenyl-H), 7.87 (s, 1H, pyrimidine-H), 7.53 (dd, 2H, *J_1_* = 5.40 Hz, *J_2_* = 3.00 Hz, phenyl-H), 7.50 (d, 2H, *J* = 8.40 Hz, phenyl-H), 7.42 (t, 2H, *J* = 9.00 Hz, phenyl-H), 4.63 (s, 2H, -SCH_2_-); ^13^C NMR (150 MHz, DMSO-*d*_6_, ppm) δ: 170.33, 167.65, 165.34, 162.88, 161.26, 159.73, 156.21 (q, *J* = 35.1 Hz), 154.28, 133.22, 131.75, 129.74, 127.63, 123.38, 121.80 (q, *J* = 273.15 Hz), 115.98, 115.82, 107.08, 37.21; HRMS (ESI) calcd for C_20_H_12_ON_4_S_2_F_4_ [M+Na]^+^: 487.02747, found: 487.02809.

Data for 2-((2-dichlorobenzyl)thio)-5-(4-((6-(trifluoromethyl)pyrimidin-4-yl)oxy)

phenyl)-1,3,4-thiadiazole (**6d**). White solid; yield 62.62%; m.p. 72−74 °C; ^1^H NMR (600 MHz, DMSO-*d*_6_, ppm) δ: 8.99 (s, 1H, pyrimidine-H), 8.04 (d, 2H, *J* = 9.00 Hz, phenyl-H), 7.88 (s, 1H, pyrimidine-H), 7.63 (d, 1H, *J* = 7.80 Hz, phenyl-H), 7.52 (t, 1H, *J* = 7.80 Hz, phenyl-H), 7.46 (t, 1H, *J* = 8.40 Hz, phenyl-H), 7.37−7.33 (m, 1H, Ph-H), 7.21 (d, 2H, *J* = 9.00 Hz, Ph-H), 4.63 (s, 2H, -SCH_2_-); ^13^C NMR (150 MHz, DMSO-*d*_6_, ppm) δ: 170.32, 168.66, 167.98, 164.79, 163.43, 161.04, 159.73, 164.72, 156.22 (q, *J* = 34.8 Hz), 154.33, 134.27, 133.90, 133.22, 132.12, 130.76, 130.56, 129.94, 129.78, 127.97, 123.38, 121.80 (q, *J* = 273.4 Hz), 116.12, 107.05, 36.22; HRMS (ESI) calcd for C_20_H_12_ON_4_S_2_ClF_3_ [M+Na]^+^: 502.99716, found: 502.99854.

Data for 2-((3-chlorobenzyl)thio)-5-(4-((6-(trifluoromethyl)pyrimidin-4-yl)oxy)

phenyl)-1,3,4-thiadiazole (**6e**). White solid; yield 54.24%; m.p. 109−111 °C; ^1^H NMR (600 MHz, DMSO-*d*_6_, ppm) δ: 8.99 (s, 1H, pyrimidine-H), 8.03 (d, 2H, *J* = 8.40 Hz, phenyl-H), 7.87 (s, 1H, phenyl-H), 7.59 (s, 1H, pyrimidine-H), 7.53 (d, 2H, *J* = 8.40 Hz, phenyl-H), 7.48 (d, 1H, *J* = 7.80 Hz, phenyl-H), 7.40 (t, 1H, *J* = 7.80 Hz, phenyl-H), 7.37 (d, 1H, *J* = 7.80 Hz, phenyl-H), 4.72 (s, 2H, -SCH_2_-); ^13^C NMR (150 MHz, DMSO-*d*_6_, ppm) δ: 170.32, 167.78, 165.13, 159.73, 156.22 (q, *J* = 35.25 Hz), 154.20, 139.70, 133.51, 130.90, 129.76, 129.42, 128.34, 128.13, 127.60, 126.73, 123.37, 121.80 (q, *J* = 272.70 Hz), 116.19, 107.06, 37.17; HRMS (ESI) calcd for C_20_H_12_ON_4_S_2_ClF_3_ [M+Na]^+^: 502.99716, found: 502.99854.

Data for 2-((4-chlorobenzyl)thio)-5-(4-((6-(trifluoromethyl)pyrimidin-4-yl)oxy)

phenyl)-1,3,4-thiadiazole (**6f**). White solid; yield 65.81%; m.p. 143−145 °C; ^1^H NMR (600 MHz, DMSO-*d*_6_, ppm) δ: 8.99 (s, 1H, pyrimidine-H), 8.03 (d, 2H, *J* = 8.40 Hz, phenyl-H), 7.59 (s, 1H, pyrimidine-H), 7.53 (d, 2H, *J* = 8.40 Hz, Ph-H),7.49 (d, 3H, *J* = 9.00 Hz, Ph-H), 7.43 (d, 2H, *J* = 8.40 Hz, Ph-H), 4.63 (s, 2H, -SCH_2_-); ^13^C NMR (150 MHz, DMSO-*d*_6_, ppm) δ: 170.32, 167.71, 165.22, 159.73, 156.21 (q, *J* = 34.95 Hz), 154.29, 136.18, 132.79, 131.50, 129.75, 129.02, 127.62, 121.80 (q, *J* = 272.85 Hz), 116.19, 107.07, 37.18; HRMS (ESI) calcd for C_20_H_12_ON_4_S_2_ClF_3_ [M+Na]^+^: 502.99716, found: 502.99854.

Data for 2-(((5-(4-((6-(trifluoromethyl)pyrimidin-4-yl)oxy)phenyl)-1,3,4-thiadiazol-

2-yl)thio)methyl)benzonitrile (**6g**). White solid; yield 60.33%; m.p. 135−137 °C; ^1^H NMR (600 MHz, DMSO-*d*_6_, ppm) δ: 8.99 (s, 1H, pyrimidine-H), 8.04 (d, 2H, *J* = 8.4 Hz, Ph-H), 7.90 (d, 1H, *J* = 8.40 Hz, phenyl-H), 7.87 (s, 1H, pyrimidine-H), 7.76 (d, 1H, *J* = 7.8 Hz, Ph-H), 7.72 (t, 1H, *J* = 7.8 Hz, Ph-H), 7.53 (t, 1H, *J* = 7.80 Hz, Ph-H), 7.50 (d, 1H, *J* = 9.00 Hz, Ph-H), 4.65 (s, 2H, -SCH_2_-); ^13^C NMR (150 MHz, DMSO-*d*_6_, ppm) δ: 170.23, 168.30, 164.02, 159.73, 156.21 (q, *J* = 34.8 Hz), 154.36, 140.49, 133.93, 133.80, 131.10, 129.81, 129.20, 127.55, 123.38, 121.81 (q, *J* = 272.85 Hz), 117.70, 112.47, 107.08, 36.41; HRMS (ESI) calcd for C_21_H_12_ON_5_S_2_F_3_ [M+Na]^+^: 494.03226, found: 494.03276.

Data for 2-((4-(trifluoromethyl)benzyl)thio)-5-(4-((6-(trifluoromethyl)pyrimidin-4-yl)

oxy)phenyl)-1,3,4-thiadiazole (**6h**). White solid; yield 34.82%; m.p. 124−126 °C; ^1^H NMR (600 MHz, DMSO-*d*_6_, ppm) δ: 8.99(s, 1H, pyrimidine-H), 8.02 (d, 2H, *J* = 8.40 Hz, phenyl-H), 7.85 (s, 1H, pyrimidine-H), 7.72−7.66 (m, 4H, phenyl-H), 7.49 (d, 2H, *J* = 8.40 Hz, Ph-H), 4.73 (s, 2H, -SCH_2_-); ^13^C NMR (150 MHz, DMSO-*d*_6_, ppm) δ: 170.30, 167.79, 165.02, 159.72, 156.23 (q, *J* = 34.95 Hz), 154.29, 142.17, 130.39, 129.73, 128.73 (q, *J* = 31.80 Hz), 127.59, 125.89, 125.53 (q, *J* = 270.15 Hz), 123.34, 121.78 (q, *J* = 272.85 Hz), 107.02, 37.18; HRMS (ESI) calcd for C_21_H_12_ON_4_S_2_F_6_ [M+Na]^+^: 537.02356, found: 537.02489.

Data for 2-((3,4-dichlorobenzyl)thio)-5-(4-((6-(trifluoromethyl)pyrimidin-4-yl)oxy)

phenyl)-1,3,4-thiadiazole (**6i**). White solid; yield 49.04%; m.p. 125−127 °C; ^1^H NMR (600 MHz, DMSO-*d*_6_, ppm) δ: 8.99 (s, 1H,pyrimidine-H), 8.02 (d, 2H, *J* = 8.4 Hz, phenyl-H), 7.85 (s, 1H, pyrimidine-H), 7.78 (d, 1H, *J* = 1.80 Hz, phenyl-H), 7.62 (d, 1H, *J* = 8.40 Hz, phenyl-H), 7.51−7.47 (m, 3H, phenyl-H), 4.64 (s, 2H, -SCH_2_-); ^13^C NMR (150 MHz, DMSO-*d*_6_, ppm) δ: 170.30, 167.84, 164.89, 159.72, 156.24 (q, *J* = 35.40 Hz), 154.30, 138.58, 131.95, 131.56, 131.48, 131.14, 130.76, 129.95, 129.74, 129.12, 127.58, 123.33, 121.79 (q, *J* = 273.60 Hz), 116.03, 107.02, 34.49; HRMS (ESI) calcd for C_20_H_11_ON_4_S_2_Cl_2_F_3_ [M+Na]^+^: 502.99716, found: 502.99854.

Data for 2-(4-((2-methyl-6-(trifluoromethyl)pyrimidin-4-yl)oxy)phenyl)-5-((2-methyl

benzyl)thio)-1,3,4-thiadiazole (**6j**). White solid; yield 60.82%; m.p. 110−112 °C; ^1^H NMR (600 MHz, DMSO-*d*_6_, ppm) δ: 8.02 (d, 2H, *J* = 9.00 Hz, phenyl-H), 7.61 (s, 1H, pyrimidine-H), 7.48 (d, 2H, *J* = 9.00 Hz, phenyl-H), 7.43 (d, 1H, *J* = 7.2 Hz, phenyl-H), 7.24−7.21 (m, 2H, phenyl-H), 7.19−7.16 (m, 1H, phenyl-H), 4.65 (s, 2H, -SCH_2_-), 2.53 (s, 3H, pyrimidine-CH_3_), 2.41 (s, 3H, phenyl-CH_3_); ^13^C NMR (150 MHz, DMSO-*d*_6_, ppm) δ: 170.19, 167.68, 167.70, 165.27, 156.41 (q, *J* = 35.25 Hz), 154.35, 137.37, 134.14, 128.64, 127.48, 126.62, 123.23, 121.81 (q, *J* = 273.00 Hz), 103.83, 36.69, 25.89, 19.27; HRMS (ESI) calcd for C_22_H_17_ON_4_S_2_F_3_ [M+Na]^+^: 497.06799, found: 497.06881.

Data for 2-((4-fluorobenzyl)thio)-5-(4-((2-methyl-6-(trifluoromethyl)pyrimidin-4-yl)

oxy)phenyl)-1,3,4-thiadiazole (**6k**). White solid; yield 49.10%; m.p. 128−130 °C; ^1^H NMR (600 MHz, DMSO-*d*_6_, ppm) δ: 8.02 (d, 2H, *J* = 8.40 Hz, phenyl-H), 7.61 (s, 1H, pyrimidine-H), 7.53 (dd, 2H, *J_1_* = 6.00 Hz, *J_2_* = 3.00 Hz, phenyl-H), 7.48 (d, 2H, *J* = 8.40 Hz, phenyl-H), 7.20 (t, 2H, *J* = 9.00 Hz, phenyl-H), 4.63 (s, 2H, -SCH_2_-), 2.52 (s, 3H, pyrimidine-CH_3_); ^13^C NMR (150 MHz, DMSO-*d*_6_, ppm) δ: 170.19, 167.67, 167.63, 165.27, 162.88, 161.26, 156.41 (q, *J* = 34.50 Hz), 154.35, 133.20, 131.74, 129.71, 127.45, 123.23, 121.80 (q, *J* = 273.15 Hz), 115.95, 115.81, 103.82, 37.23, 25.89; HRMS (ESI) calcd for C_21_H_14_ON_4_S_2_F_4_ [M+Na]^+^: 501.04266, found: 501.04374.

Data for 2-((2-chlorobenzyl)thio)-5-(4-((2-methyl-6-(trifluoromethyl)pyrimidin-4-yl)

oxy)phenyl)-1,3,4-thiadiazole (**6l**). White solid; yield 68.66%; m.p. 110−112 °C; ^1^H NMR (600 MHz, DMSO-*d*_6_, ppm) δ: 8.03 (d, 2H, *J* = 9.0 Hz, phenyl-H), 7.65 (dd, 1H, *J_1_* = 1.8 Hz, *J_2_* = 5.4 Hz, phenyl-H), 7.60 (s, 1H, pyrimidine-H), 7.52 (dd, 2H, *J_1_* = 1.80 Hz, *J_2_* = 6.00 Hz, phenyl-H), 7.48 (d, 2H, *J* = 9.0 Hz, phenyl-H), 7.38−7.33 (m, 2H, phenyl-H), 4.72 (s, 2H, -SCH_2_-), 2.53 (s, 3H, pyrimidine-CH_3_); ^13^C NMR (150 MHz, DMSO-*d*_6_, ppm) δ: 170.18, 169.68, 168.00, 164.72, 156.42 (q, *J* = 34.80 Hz), 154.38, 134.21, 133.90, 132.09, 130.36, 130.13, 129.74, 127.95, 127.42, 123.22, 121.81 (q, *J* = 272.70 Hz), 103.83, 36.22, 25.89; HRMS (ESI) calcd for C_21_H_14_ON_4_S_2_ClF_3_ [M+Na]^+^: 517.01379, found: 517.01419.

Data for 2-((3-chlorobenzyl)thio)-5-(4-((2-methyl-6-(trifluoromethyl)pyrimidin-4-yl)

oxy)phenyl)-1,3,4-thiadiazole (**6m**). White solid; yield 49.05%; m.p. 128−131 °C; ^1^H NMR (600 MHz, DMSO-*d*_6_, ppm) δ: 8.03 (d, 2H, *J* = 9.00 Hz, phenyl-H), 7.65 (dd, 1H, *J_1_* = 1.80 Hz, *J_2_* = 5.40 Hz, phenyl-H), 7.61 (s, 1H, pyrimidine-H), 7.53 (dd, 2H, *J_1_* = 1.80 Hz, *J_2_* = 6.00 Hz, phenyl-H), 7.48 (d, 2H, *J* = 9.00 Hz, phenyl-H), 7.38−7.33 (m, 2H, phenyl-H), 4.72 (s, 2H, -SCH_2_-), 2.53 (s, 3H, pyrimidine-CH_3_); ^13^C NMR (150 MHz, DMSO-*d*_6_, ppm) δ: 170.18, 169.68, 169.02, 164.73, 156.41 (q, *J* = 34.80 Hz), 154.39, 134.22, 133.90, 132.10, 130.37, 130.13, 129.75, 127.96, 127.43, 123.23, 121.81 (q, *J* = 273.45 Hz), 103.84, 36.24, 25.90; HRMS (ESI) calcd for C_21_H_14_ON_4_S_2_ClF_3_ [M+Na]^+^: 517.01282, found: 517.01419.

Data for 2-((4-dichlorobenzyl)thio)-5-(4-((2-methyl-6-(trifluoromethyl)pyrimidin-

4-yl)oxy)phenyl)-1,3,4-thiadiazole (**6n**). White solid; yield 49.48%; m.p. 105−107 °C; ^1^H NMR (600 MHz, DMSO-*d*_6_, ppm) δ: 8.01 (d, 2H, *J* = 8.40 Hz, phenyl-H), 7.60 (s, 1H, pyrimidine-H), 7.53 (d, 1H, *J* = 8.40 Hz, phenyl-H), 7.46 (d, 1H, *J* = 8.40 Hz, phenyl-H), 7.42 (d, 2H, *J* = 9.00 Hz, phenyl-H), 4.63 (s, 2H, -SCH_2_-), 2.52 (s, 3H, pyrimidine-CH_3_); ^13^C NMR (150 MHz, DMSO-*d*_6_, ppm) δ: 170.18, 169.67, 167.72, 165.13, 156.42 (q, *J* = 34.50 Hz), 154.35, 136.17, 132.79, 130.47, 129.70, 129.01, 127.44, 123.21, 121.81 (q, *J* = 272.70 Hz), 103.80, 37.20, 25.88; HRMS (ESI) calcd for C_21_H_14_ON_4_S_2_ClF_3_ [M+Na]^+^: 517.01282, found: 517.01419.

Data for 2-((2,4-dichlorobenzyl)thio)-5-(4-((2-methyl-6-(trifluoromethyl)pyrimidin-

4-yl)oxy)phenyl)-1,3,4-thiadiazole (**6o**). White solid; yield 49.90%; m.p. 143−145 °C; ^1^H NMR (600 MHz, DMSO-*d*_6_, ppm) δ: 8.03 (d, 2H, *J* = 9.00 Hz, phenyl-H), 7.69 (d, 1H, *J* = 1.80 Hz, phenyl-H), 7.67 (d, 1H, *J* = 8.40 Hz, phenyl-H), 7.61 (s, 1H, pyrimidine-H), 7.48 (d, 2H, *J* = 8.40 Hz, phenyl-H), 7.45 (dd, 1H, *J_1_* = 1.80 Hz, *J_2_* = 6.60 Hz, phenyl-H), 4.70 (s, 2H, -SCH_2_-), 2.52 (s, 3H, pyrimidine-CH_3_); ^13^C NMR (150 MHz, DMSO-*d*_6_, ppm) δ: 170.18, 169.67, 168.16, 164.41, 156.41 (q, *J* = 34.80 Hz), 154.40, 134.87, 133.93, 133.61, 133.30, 129.76, 129.60, 128.08, 127.40, 123.23, 121.81 (q, *J* = 272.85 Hz), 103.84, 35.58, 25.89; HRMS (ESI) calcd for C_22_H_14_ON_5_S_2_ClF_3_ [M+Na]^+^: 508.04819, found: 508.04841.

Data for 2-(4-((2-methyl-6-(trifluoromethyl)pyrimidin-4-yl)oxy)phenyl)-5((2(trifluoro

methyl)benzyl) thio)-1,3,4-thiadiazole (**6p**). White solid; yield 78.02%; m.p. 102−104 °C; ^1^H NMR (600 MHz, DMSO-*d*_6_, ppm) δ: 8.03 (d, 2H, *J* = 8.40 Hz, phenyl-H), 7.81 (t, 2H, *J* = 8.40 Hz, phenyl-H), 7.71 (t, 1H, *J* = 7.20 Hz, phenyl-H), 7.60 (s, 1H, pyrimidine-H), 7.56 (t, 1H, *J* = 7.8 Hz, Ph-H), 7.48 (d, 2H, *J* = 9.0 Hz, phenyl-H), 4.81 (s, 2H, -SCH_2_-), 2.53 (s, 3H, pyrimidine-CH_3_); ^13^C NMR (150 MHz, DMSO-*d*_6_, ppm) δ: 170.17, 169.67, 168.10, 164.52, 156.42 (q, *J* = 34.65 Hz), 154.41, 134.72, 133.52, 132.53, 129.76, 129.13, 127.84 (q, *J* = 29.70 Hz), 127.39, 126.88 (q, *J* = 5.55 Hz), 126.65 (q, *J* = 272.40 Hz), 123.23, 121.80 (q, *J* = 273.00 Hz), 103.83, 34.90, 25.88; HRMS (ESI) calcd for C_22_H_14_ON_4_S_2_F_6_ [M+Na]^+^: 551.03992, found: 551.04054.

Data for 2-(4-((2-methyl-6-(trifluoromethyl)pyrimidin-4-yl)oxy)phenyl)-5-((4-

(trifluoromethyl) benzyl)thio)-1,3,4-thiadiazole (**6q**). White solid; yield 68.26%; m.p. 95−96 °C; ^1^H NMR (600 MHz, DMSO-*d*_6_, ppm) δ: 8.01 (d, 2H, *J* = 8.40 Hz, phenyl-H), 7.73−7.71 (m, 4H, phenyl-H), 7.60 (s, 1H, pyrimidine-H), 7.48 (d, 2H, *J* = 9.00 Hz, phenyl-H), 4.73 (s, 2H, CH_2_), 2.52 (s, 3H, pyrimidine-CH_3_); ^13^C NMR (150 MHz, DMSO-*d*_6_, ppm) δ: 170.18, 169.67, 167.84, 164.96, 156.42 (q, *J* = 34.65 Hz), 154.37, 142.19, 130.39, 132.17 (q, *J* = 30.60 Hz), 129.71, 128.52, 127.41, 125.88, 125.54 (q, *J* = 270.30 Hz), 123.21, 121.81 (q, *J* = 273.45 Hz), 116.18, 103.80, 34.90, 25.88; HRMS (ESI) calcd for C_22_H_14_ON_4_S_2_F_6_ [M+Na]^+^: 551.03973, found: 551.04054.

Data for 2-((2,3-dichlorobenzyl)thio)-5-(4-((2-methyl-6-(trifluoromethyl)pyrimidin-

4-yl)oxy) phenyl)-1,3,4-thiadiazole (**6r**). White solid; yield 56.75%; m.p. 123−125 °C; ^1^H NMR (600 MHz, DMSO-*d*_6_, ppm) δ: 8.01 (d, 2H, *J* = 8.40 Hz, phenyl-H), 7.62 (t, 2H, *J* = 6.60 Hz, phenyl-H), 7.62 (d, 2H, *J* = 8.40 Hz, Ph-H), 7.59 (s, 1H, pyrimidine-H), 7.48 (d, 2H, *J* = 8.40 Hz, phenyl-H), 7.37 (t, 1H, *J* = 7.80 Hz, phenyl-H), 4.76 (s, 2H, -SCH_2_-), 2.51 (s, 3H, pyrimidine-CH_3_); ^13^C NMR (150 MHz, DMSO-*d*_6_, ppm) δ: 170.17, 169.67, 168.15, 164.40, 156.42 (q, *J* = 34.65 Hz), 154.40, 137.04, 132.66, 131.95, 130.65, 129.74, 128.74, 127.39, 123.21, 121.80 (q, *J* = 273.30 Hz), 103.80, 35.87, 25.88; HRMS (ESI) calcd for C_21_H_13_ON_4_S_2_Cl_2_F_3_ [M+Na]^+^: 550.97461, found: 550.97521.

Data for 2-(((5-(4-((2-methyl-6-(trifluoromethyl)pyrimidin-4-yl)oxy)phenyl)-

1,3,4-thiadiazol-2-yl) thio)methyl)benzonitrile (**6s**). White solid; yield 80.58%; m.p. 111−112 °C; ^1^H NMR (600 MHz, DMSO-*d*_6_, ppm) δ: 8.02 (d, 2H, *J* = 9.00 Hz, phenyl-H), 7.90 (d, 1H, *J* = 7.20 Hz, phenyl-H), 7.76 (d, 1H, *J* = 7.80 Hz, phenyl-H), 7.72 (t, 1H, *J* = 7.80 Hz, phenyl-H), 7.61 (s, 1H, pyrimidine-H), 7.53 (t, 1H, *J* = 7.20 Hz, phenyl-H), 7.48 (d, 1H, *J* = 8.40 Hz, phenyl-H), 4.80 (s, 2H, -SCH_2_-), 2.41 (s, 3H, pyrimidine-CH_3_); ^13^C NMR (150 MHz, DMSO-*d*_6_, ppm) δ: 170.18, 167.67, 168.33, 163.95, 156.41 (q, *J* = 34.50 Hz), 154.43, 140.49, 133.93, 133.80, 131.08, 129.78, 129.20, 127.37, 123.23, 121.81 (q, *J* = 273.00 Hz), 117.70, 112.48, 103.84, 36.43, 25.89; HRMS (ESI) calcd for C_21_H_13_ON_4_S_2_Cl_2_F_3_ [M+Na]^+^: 550.97437, found: 550.97521.

Data for 2-((3,4-dichlorobenzyl)thio)-5-(4-((2-methyl-6-(trifluoromethyl)pyrimidin-4-

yl)oxy)phenyl) -1,3,4-thiadiazole (**6t**). White solid; yield 67.5%; m.p. 95−96 °C; ^1^H NMR (600 MHz, DMSO-*d*_6_, ppm) δ: 8.03 (d, 2H, *J* = 8.40 Hz, phenyl-H), 7.69 (d, 1H, *J* = 1.80 Hz, phenyl-H), 7.62 (d, 1H, *J* = 8.40 Hz, phenyl-H), 7.60 (s, 1H, pyrimidine-H), 7.51 (dd, 1H, *J_1_* = 2.40 Hz, *J_2_* = 6.00 Hz, phenyl-H), 7.48 (d, 2H, *J* = 9.00 Hz, phenyl-H), 4.64 (s, 2H, -SCH_2_-), 2.52 (s, 3H, pyrimidine-CH_3_); ^13^C NMR (150 MHz, DMSO-*d*_6_, ppm) δ: 170.18, 169.67, 167.90, 164.84, 156.41 (q, *J* = 34.50 Hz), 154.37, 138.60, 131.56, 131.47, 131.15, 130.74, 129.96, 129.73, 127.40, 123.21, 121.81 (q, *J* = 272.85 Hz), 103.83, 35.50, 25.89; HRMS (ESI) calcd for C_21_H_13_ON_4_S_2_Cl_2_F_3_ [M+Na]^+^: 550.97461, found: 550.97508.
